# Relating to the Speaker behind the Voice: What Is Changing?

**DOI:** 10.3389/fpsyg.2018.00011

**Published:** 2018-01-25

**Authors:** Felicity Deamer, Mark Hayward

**Affiliations:** ^1^Department of Philosophy, Durham University, Durham, United Kingdom; ^2^School of Psychology, University of Sussex, Falmer, United Kingdom

**Keywords:** AVH, voice hearing, relating therapy, avatar therapy, communication

## Abstract

We introduce therapeutic techniques that encourage voice hearers to view their voices as coming from intentional agents whose behavior may be dependent on how the voice hearer relates to and interacts with them. We suggest that this approach is effective because the communicative aspect of voice hearing might fruitfully be seen as explanatorily primitive, meaning that the agentive aspect, the auditory properties, and the intended meaning (interpretation) are all necessary parts of the experience, which contribute to the impact the experience has on the voice hearer. We examine the experiences of a patient who received Relating Therapy, and explore the kinds of changes that can result from this therapeutic approach.

## Introducing relational therapies

Auditory Verbal Hallucinations (AVH)—the experience of hearing a voice(s) of someone/thing that does not seem to be physically present—can have a devastating effect on patients' lives due to high levels of distress (Birchwood and Chadwick, 1997), depression (Birchwood et al., 2004), impaired social functioning (Favrod et al., 2004), delayed recovery (de Jager et al., 2015), and an increased risk of suicide (Kjelby et al., 2015). More than 200,000 patients in England and Wales hear distressing voices in the context of a psychosis diagnosis (Royal College of Psychiatrists, 2014), and this experience is also common in other psychiatric disorders (Sommer et al., 2012). Patients who hear distressing voices report the reduction of distress to be a key priority for treatment, over, and above outcomes such as social and occupational recovery and general wellbeing (Strauss, 2016).

The National Institute for Health and Care Excellence (NICE) recommend antipsychotic medication and Cognitive Behavior Therapy for Psychosis (CBTp) for the treatment of distressing voices (National Collaborating Centre for Mental Health, 2014). Despite evidence for its benefits, antipsychotic medication is often not fully effective (Morrison et al., 2012) and 40–50% of patients are non-adherent (Lacro et al., 2002). CBTp has evidence from 12 meta-analyses which suggest small to moderate effects, and a mean effect size of *d* = 0.44 on AVH outcomes more specifically (van der Gaag et al., 2014). However, despite successfully targeting key mechanisms of appraisals about the power of the AVH, CBTp has not consistently reduced associated distress (Mawson et al., 2010). A review of 16 studies of CBTp that included a specific measure of AVH reported only two studies that showed a significant reduction in distress—and in both cases this effect was not maintained at follow-up (McCarthy-Jones et al., 2015).

What can account for this apparent discrepancy between the effectiveness of CBTp for AVH, and its limited impact upon distress? One suggestion is that the links between appraisals of AVH and distress are more complex than originally thought. Researchers have suggested that interpersonal/social schema may be exerting an influence (Paulik, 2012; Hayward et al., 2014), and this has prompted a shift from conceptualizing an AVH as a sensory stimulus that the hearer holds *beliefs about*, to an AVH as a person-like stimulus which the hearer has a *relationship with* (Hayward et al., 2011)^1^. Empirical studies have generated data using three different interpersonal theories to support this conceptualization of patients having relationships with their AVH (Benjamin, 1989; Birchwood et al., 2000; Sorrell et al., 2010), and these relationships have been found to share similarities with social relationships (Hayward, 2003; Birchwood et al., 2004). Furthermore, relationships with voices can be positive in non-patients (Jackson et al., 2011), including spiritualist voice hearers, and do not seem to change spontaneously in patients (Hartigan et al., 2014). Collectively, this data seems to provide a mandate for developing therapies that seek to modify the relating of patients within difficult relationships—with both AVH and other people.

At least three therapies are being developed that attempt to modify the way that patients respond relationally to distressing and negative voices. The Voice Dialogue approach conceptualizes distressing and negative voices as a dissociated “part” of the self and seeks to facilitate constructive “live” dialogue between the patient and the AVH (Corstens et al., 2012). AVATAR therapy takes a different approach; rather than seeking to modify dialogues with AVH that are talking “live,” a visual depiction of the AVH is created and displayed on a computer screen and the patient is coached to respond assertively to this avatar (whose responses are generated by the therapist in a different room; Craig et al., 2018). Relating Therapy takes an even more emotionally detached approach to modifying relationships by using experiential role-plays to practice articulating assertive responses to the typical utterances of the AVH (or the social other with whom the patient is in a difficult relationship; Hayward et al., 2017). Relating Therapy and its outcomes will be explored in greater detail below prior to a linguistically-informed consideration of what might be changing within relationships to generate the beneficial outcomes.

## What happens within relating therapy?

The primary premise of Relating Therapy (RT) is that the view of the patient is not articulated within difficult relationships—be they with negative and distressing voices or other people. Consequently, within RT the patient is taught how to articulate their view in an assertive and respectful manner. This assertiveness training is preceded by two prior phases of therapy that seek to: (1) raise awareness of the role that the patient currently plays within difficult relationships, and how this role may be maintaining difficulty; and (2) explore and identify patterns in the relational history of the patient, in a way that normalizes the current passive and/or aggressive responding and locates it within a broader developmental and relational context.

Once the patient has a sense of their non-assertive relating and its history, pervasiveness and consequences, a specific difficult relationship is chosen (with an AVH or other person) and explored in greater detail. An emphasis is placed upon the utterances of the AVH/other within the chosen relationship (which in the case of AVH are often quite repetitive and relate to criticism and commands), and a specific utterance is selected. The patient is invited to explore their typical emotional, behavioral and relational responses to this utterance, and most importantly—to explore its accuracy. If the AVH/other has said “you're useless,” the patient is assisted by the therapist to use CBTp techniques to collaboratively consider all of the available evidence that does and does not fit with this utterance. If, after reviewing the evidence, the patient concludes that the utterance is not entirely accurate and the they hold a (slightly) different view, the focus turns to ways of articulating this differing view.

The expression of a different view is initially constructed verbally on paper, and might be something like “I hear what you're saying and I do feel useless some of the time; but at other times I can do things well, and last week I received thanks from a friend for helping them.” Having constructed this verbal and assertive response, RT moves into its experiential work where the patient is invited to practice assertive responding within roleplays. The roleplays are brief and multiple, and the patient can play themselves or the AVH/other (with the therapist taking the other role), and move between the roles. Initially, the emphasis is upon saying the assertive words, and extensively reflecting upon this experience and revising the response. When the response can be articulated assertively, the focus moves to non-verbal communication—how to look and feel assertive, as well as sounding assertive. Subsequently, the focus moves toward remaining assertive during increasingly longer roleplays, and continuing to draw upon evidence to support the view that is being articulated.

Within RT the chosen relationship is not the sole focus of the experiential work. The patient's experience between sessions may foreground another difficult relationship, and this can be the focus of therapy for one or more sessions; thereby corroborating the emphasis placed upon learning adaptive ways of relating that can be generalized to all difficult relationships. It also worth noting that RT does not make assumptions about how the AVH/other will respond to the new-found assertive relating of the patient. The patient is invited to focus exclusively upon their own relating—and see what happens next. In this sense, the patient is discouraged from arguing with and trying to discredit the view of the AVH/other.

RT was initially developed and evaluated quantitatively and qualitatively through a case series (Hayward et al., 2009; Hayward and Fuller, 2010). All five patients completed the therapy that was described as intuitively appealing. Within a subsequent pilot RCT, RT was delivered to 14 patients (with varying diagnoses) and their outcomes were compared with 15 patients who received only their usual care. Attrition was minimal and outcomes were very encouraging, with a large between-groups effect found for the primary outcome of AVH-distress, that was maintained at follow-up (Hayward et al., 2017). Medium to large effects were reported for the secondary outcomes of subjective recovery and depression, and a small to medium effect was found for social relating—all maintained at follow-up.

This raises some important questions; What is it about AVH that means that relational therapies, such as Relating Therapy, are generating such positive outcomes? What changes might result from the therapy, and how do these changes generate beneficial outcomes?

## The communicative aspect of AVH

### Beyond the auditory: a starting point

Traditionally, aetiological models of AVH have sought to uncover the cognitive mechanisms or processes (i.e., intrusions from memory, misattributed inner speech, or spontaneous activation of the auditory cortex) that might afford the possibility of hearing a voice in the absence of any such auditory stimulus. These audio-centric approaches, which have focused on the question of “how” (physiologically) voice hearing can come about at all, have failed to account for why AVH have the content and properties that they do, why they are often so distressing, and what other factors (both psychological and environmental) might play a part in their occurrence. As well as limiting their capacity to shed light on the phenomenology of voice hearing, they say very little about why recent developments in therapeutic techniques, in which the interpersonal nature of AVH is placed at the fore, are generating positive outcomes. Recently, however, there has been an important shift in focus, away from the auditory (how it is possible to hear something in the absence of any auditory stimulus), and onto the agentive aspect of the voice hearing experience; the sense that the voice heard is coming from a particular individual, often with identifiable intentions, characteristics, attitudes, and beliefs. Wilkinson and Bell (2014) argue that it is the representation of agency, rather than the auditory properties of AVH, that is central to the phenomenon.

This new approach to thinking about voice hearing is, at least in part, motivated by findings that suggest that not all AVH are in fact experienced as auditory (and even when they are, it is not clear that it is this aspect of the experience that is the primary cause of distress), and that many voice hearers report that a representation of the speaker behind the voice that they hear constitutes a more or less significant aspect of their experience. In this section, we argue that although this shift in emphasis brings with it a richer understanding of voice hearing, both phenomenologically and aetiologically, by giving a different but similarly singular aspect of the voice hearing experience explanatory primacy (i.e., agentive properties rather than auditory properties), some of the more fine-grained features of the agentive aspect of the experience remain unexplained; agency is often only sparsely represented (as Wilkinson and Bell acknowledge), and when it is represented, it (typically) only manifests itself in a specific and narrow sense, as a speaker behind the voice, and in the case of distressing voices, as a malicious speaker behind the voice. We put forward an importantly different approach to understanding voice hearing, in which the communicative nature of voice hearing is taken to be explanatorily prior (needs explaining first and foremost), and as such, the finer details of the agentive and auditory properties of the experience are accounted for, and some light is shed on the distressing nature of many voice hearing experiences.

### Wilkinson and bell (2014)

Wilkinson and Bell don't just draw attention to the representation of agency in AVH, they argue that it is central to the phenomenon, and is causally connected to other properties of AVH. As they point out, proponents of traditional approaches might claim that agency is represented in AVH because it sounds like the voice of an individual, but Wilkinson and Bell claim that the finer details of how the voice sounds and what it says are determined by who is speaking, not the other way around. Their claims are motivated by the heterogeneity of AVH; Although many voice hearers report hearing a sound which is unambiguously a voice, with all the relevant acoustic properties (Cho and Wu, 2013), it has been emphasized that AVH can be devoid of any auditory properties “an experience of receiving a communication without any sensory component” (Frith, 1992, p.73), causing some to suggest that the label “Auditory Verbal Hallucination” is something of a misnomer (Moritz and Larøi, 2008, p. 104). Wilkinson and Bell ague that “soundless voices” have been recorded and discussed within the literature for more than 100 years (Bleuler, 1911, 1950; Jones, 2010; Larøi et al., 2012), so it is odd that current aetiological models should completely overlook this by focusing on explaining why somebody might hear something in the absence of an auditory stimulus. Instead, they propose that it is the subjective experience of an agent which comes closer to unifying instances of AVH, and thus needs explaining.

According to Wilkinson and Bell, voice hearing experiences are afforded by (among other things) our natural (adaptive) propensity as social beings, to represent and track other (often specific) social agents; ones that have some significance to us as individuals. Being predisposed to distinguish the animate from the inanimate, and to recognize that the former behave in goal-directed ways in accordance with their beliefs and desires, leads to us to have a “hyperactive agency detection” (Guthrie, 1980; Atran and Norenzayan, 2004; Barrett, 2004). Moreover, being able to keep track of specific individuals who we might encounter again and being able to associate those individuals with risks will have been highly adaptive, and is now “a vital component of healthy social cognition, [and] of successful interaction in a world that is populated by other agents that we encounter and re-encounter.” Some individuals that we encounter in our lives are likely to remain remarkably salient due to the potential threat associated with them, and it is this, together with our hyperactive agency detection, that Wilkinson and Bell think is behind voice hearing.

“*The representation of the man who abused you as a child is the most active, the one you most fear. Thus, to turn the standard explanatory order on its head: the voice has the properties that it has because it is represented as the voice of a given individual, rather than the other way around. For example, it is a deep, gruff voice, because it is represented as my stepfather's voice, rather than the other way around*.” (pg. 117)

We are very sympathetic to this approach as it clearly comes much closer than standard etiological models of AVH to accounting for the variety within AVH. Wilkinson and Bell rightly see the significance and importance of the agentive aspect of AVH, and put forward a plausible story for how the representation of agency could be at the center of the etiology of the experience. By shifting focus from the auditory to the agentive aspect of voice hearing, their account is more in line with current effective therapeutic techniques, which have recognized that at the center of most voice hearing experiences is the perception of a malevolent agent who preys on voice hearers' low self-esteem. However, we feel that Wilkinson and Bell's approach leaves some unanswered questions. Despite noting the communicative aspect of voice hearing “these agents are most often perceived as making coherent communicative speech acts (McCarthy-Jones et al., 2014) and are experienced as interacting with the voice-hearer” their approach says nothing about why that might be. Likewise, although Wilkinson and Bell acknowledge that agency is often sparsely represented, they do not elaborate on why (if agency is seen to be explanatorily primitive) that might be the case. When agency is represented, it typically only manifests itself in a narrow sense; as a speaker behind the voice. In the case of distressing voices, in an even more narrow sense; a malevolent speaker behind the voice. We think that failing to focus on the more specific, communicative nature of the agency representation in voice hearing, renders Wilkinson and Bell's account limited in its capacity to fully explain the distressing nature of voices, and why current relational therapies are proving to be so effective.

Building on earlier work (Deamer and Wilkinson, 2015), we argue that in order to gain a better phenomenological understanding of voice hearing, to understand why voices can be so distressing, and to understand why the relational therapies discussed in this paper are generating positive outcomes, we must see the communicative acts represented in AVH as central to the phenomenon, and we must start by trying to explain them. We propose that the representation of agency and the auditory (voice-like) properties are present in so far as they are necessary dimensions of a communicative act (e.g., an utterance). By re-characterizing voice hearing as primarily communicative, the representation of agency is rightfully still understood to be a significant part of the experience, but it is the communicative acts that the agent is performing that we propose have the most explanatory power. As Wilkinson and Bell acknowledge, agents are not significant to us in and of themselves, they are significant in so far as they might be a threat to us (for example). They are a threat to us on the basis of their mental states and their actions (communicative or otherwise) that they perform on the basis of those mental states. And when they lack a physical presence, the most threatening actions at their disposal are communicative.

### The communicative act as explanatorily prior

We suggest that if you take the communicative act to be explanatorily prior, then you necessarily explain the presence of the agentive (however sparsely represented) and the auditory properties of the experience. An utterance, or any other communicative act, is a vehicle for information (e.g., opinions, beliefs, intentions), and is necessarily performed by an agent, which, we suggest, is why so many AVH are experienced as coming from specific agents, but also why agency is often only sparsely represented (it's part of the experience only in so far as it has to be).

Wilkinson and Bell's shift in focus away from the auditory aspects of voice hearing nicely explains why some voice hearing experiences might be devoid of auditory properties; they are not primarily auditory experiences. It is less clear how their focus on agent representation, alone, can explain the content of such “soundless voices” “an experience of receiving a communication without any sensory component” (Frith, 1992, p.73). As Wilkinson and Bell themselves acknowledge “It seems clear that subjects can undergo profound experiences of being spoken to, or communicated with, without the presence of any auditory phenomenology” (Wilkinson and Bell, p.109), but as they make clear, these are experiences of being communicated with or spoken to, not soundless experiences of passive agents. We suggest that voice hearing is fundamentally an experience of a communicative act, and that is why both agentive and auditory properties can be represented to a greater or lesser extent from case to case; what unifies voice hearing experiences is the communicative content (the beliefs/opinions/attitudes of the speaker behind the voice). Wilkinson and Bell refer to voice hearing in deaf subjects by way of illustrating the need to shift explanatory attention away from the auditory. Atkinson (2006) suggests that deaf voice hearers don't actually “hear” a voice at all; they just have a propensity to describe their experiences this way because it is expedient to do so in an audio-centric world. Atkinson refers to Thacker's (1994) reports, which illustrate that deaf individuals actually “claimed they were lip-reading a vague visual percept, but could not clearly see a face, or….felt that they were being finger-spelled to by a persecutor but were unable to see the hands distinctly” (Atkinson, 2006, p. 703). What these reports clearly demonstrate is that what unifies “voice hearing” in deaf and hearing people is the experience of being communicated with, or of perceiving a communicative act. It is largely irrelevant whether that act is spoken or signed (or whether there are auditory or visual properties or not), some content is conveyed.

We are not suggesting that there aren't any experiences which might previously have been included under the umbrella “AVH” which have no communicative content, in which there is no communicative agent with the intention to communicative something. However, these experiences are much less common and they might usefully be thought of as importantly different, and thus receptive to different treatments (we will return to this later).

### Therapeutic implications: not just any agent…a communicative agent

On our view, it is precisely because the malicious communicative intentions are central to the hearing of distressing negative voices that it is effective to make those intentions the primary focus of therapy, as in Relating Therapy. The therapy assists the patient to become less of an easy target for someone who “has it in for them.” It encourages them to value and stand-up for their (differing) opinion, in a manner that can enhance self-esteem. Relating Therapy (and other relational therapies) embraces the communicative aspect of the voice-hearing experience, and encourages voice hearers to view their voices as coming from communicative agents whose communicative behavior is (to a certain extent) dependent on how the voice hearer perceives and carries themselves (their level of self-esteem), and how they relate to the voice (how assertive they are).

Making the communicative content a constitutive feature of many voice hearing experiences, not only serves to illustrate how and why relational therapies might be effective for those voices, but is also suggestive of why such therapies might be entirely ineffective for other voices. As we mentioned above, there are likely to be a number of AVHs which have no communicative content, and as such would have no “speaker behind the voice.” Thus, attempting to enter into a dialogue of any sort with an absent agent will at best be futile, and at worst, potentially distressing and harmful for the patient. This kind of AVH might be equally as distressing as the communicative kind might be, but the distress will have a different cause; perhaps the intrusive and/or repetitive nature of experience, for example. And what about ‘pseudo-hallucinations’ that are perceived to be coming from “inside the head”—are these types of voice hearing experiences suitable for relational therapies? Such voices might be perceived as malevolent in so much as they are an ‘echo’ of previous adversity that is likely to have included the malevolent intentions/actions of other people. In this sense, the act of communication is between the patient and the echoes of their past experience that have left an enduring mark on their esteem—and the “speaker” is a representation of (possibly multiple) adversities. The extent to which “pseudo” voices are suitable for relationally-based therapies is likely to vary between patients, and highlights how critically important it is to ascertain the fine-grained features of a patient's voice hearing experiences during an initial assessment; what is the linguistic content? Is there a speaker behind the voice? Is that speaker malevolent? What are their intentions? In what way are the voices distressing?

### What changes might we expect to result from relating therapy?

If we view the communicative content as both fundamental to the voice-hearing experience and as the target of relational therapies, how might AVHs change as a result of such therapeutic interventions?

Initiating change in the way the patient relates to their voice, as relational therapies do, might ultimately lead to changes in the dynamic between the patient and their voice(s), albeit after some possible increases in distress that might occur through the process of change; the patient might display increasingly assertive behavior, which might mean that they perceive the voice to be less dominant. It might also trigger changes in the perception of the voice's attitude; the voice might appear less malicious and threatening, as the patient realizes that they have more control over their experiences than they initially thought. These changes will be relative to how the patient sees themselves i.e., “I have some control and worth, and no longer experience your commands and criticisms as the whole story/truth!” As a result of these changes, we might expect to see a reduction in the distress caused by the voice(s).

Is it plausible that the actual content of the voice(s) (what is said) might change as a result of therapy? In some individuals, this might be the case. However, we suggest that for many hearers of distressing voices, the negative content is perhaps a necessary part of the experience. The agent represented is necessarily a communicative agent who “has it in for them.” These individuals (who hear distressing voices) likely hear voices in so far as they are negative, so their experiences are unlikely to become altered to the point of becoming positive. The conditions under which the voices occur and/or the function they serve are unlikely to persist if the psychological state of the patient has been altered significantly by therapy. Relational Therapies are more likely to result in a reduction in the frequency of the voice(s) (i.e., they're around much less often) and/or the distress caused (i.e., they bother me less) than in any tangible change in “what the voices say.”

The next and final section is dedicated to exploring data from an exit interview with a patient who received Relating Therapy within the Sussex Voices Clinic—a specialist outpatient service within an NHS Mental Health Trust (https://www.sussexpartnership.nhs.uk/sussex-voices-clinic) The patient's descriptions of what's changing during and after therapy provide evidence of the kinds of changes a patient can experience, as a result of engaging in Relating Therapy. We examine the exit interview data through the communication centered framework we have put forward above.

## What can change as a result of relating therapy?

Jude heard one dominant female voice (“like my mum”) and a group of other male voices (“minions” to the female voice). They^2^ received eight sessions of Relating Therapy as part of a pathway of evidence-based interventions within a secondary care mental health service within the UK National Health Service. Jude's post-therapy assessment suggested that positive changes had occurred. Consequently, they were invited to participate in an exit interview to try and capture some of the subtlety of these changes. The interview was conducted by a research assistant who was independent of the therapy process.

### Changes in yourself

Jude reported a significant improvement in their general mental health, post therapy. When asked about the state of their mental health at the beginning of therapy, they said “I think when I first started the therapy [the voices] were quite bad,” “I was in a sort of dip.” They described their mental health, post therapy, as “…quite good,” and said “my voices are much easier to deal with at the moment. The stresses I do have at the moment, I know that I just need to be assertive with them, which is so helpful.”

Jude attributes this improvement in their mental health to Relating Therapy encouraging them to be more assertive. They said “the main thing I got out of [Relating Therapy] is learning how to be assertive with my voices and with other people,” “It helped me to see things differently,” “I think I have become more assertive,”

Relating Therapy has made Jude realize that it is only they themself that can control how they behave, “it doesn't matter what the other person does, it is how I conduct myself,” and that being assertive with family members can help maintain relational skills, which help with relating to voices, “because I'm practicing [being assertive] in everyday life, when the voices aren't around as much at the moment, when they do come back I'm still used to being assertive, so I can be assertive with [the voices].” Jude identifies that it is this increase in assertive skills “that's been fundamental in changing how [they] relate to the voices.”

Post Relating Therapy, Jude feels that they are reacting differently to their voices, and are more comfortable engaging with them on their own terms, “[before], I was pretending that I was ignoring them…actually I was taking on board everything they were saying….I felt like I'd failed when I shouted at them….but, I got a new perspective out of [Relating Therapy].” Jude particularly identifies the role play aspect of the therapy as having played a significant role in their new-found relationship with their voices, “[the role play] puts the theory into practice….actually demonstrating how to do it, and what to say….was so helpful,” “I would never have thought to see it from the voice's point of view [before doing the role play],” “I was seeing that [when I was in the role of the voice] there was nothing I could do to combat the assertiveness”

Jude comments on a shift in power dynamics in their relationship with their voices, as a result of therapy, “Although they're not around as much now, even when they were around more [post therapy], they weren't bothering me as much….I felt they couldn't touch me.” This is in stark contrast to how they felt before Relating Therapy, “[before] it was pretty all consuming. I felt like I was being completely controlled.” For Jude, prior to accessing Relating Therapy, they lived in fear of the voices worsening in frequency and content, “I was on tenterhooks waiting for the voices to get really bad,” “I knew that when that happened everything would fall apart.”

Not only has Jude' relationship with their voices become less negative, the negative impact that the voices have on them has lessened, “my voices tell me to do hideous things and they predict the future about what is going to happen to me, and [before Relating Therapy] I absolutely believed them that that was what was going to happen,” “[now] I don't feel like they're going to completely control me”

Jude talks of increased confidence and a new-found belief in what they are saying, “When I can be assertive with somebody, it puts confidence in my abilities” and “when I'm being assertive, I really believe in what I'm saying.” In short, Jude reports improvements in them self; more specifically, in their overall mental health, their self-esteem and in their relationship with their voice.

### Changes in your voice(s)

Jude notes a significant reduction in the frequency of their voices “The voices aren't around as much at the moment,” “I hear them, maybe, every other day.” They elaborates on this, by explaining what their voices were like before accessing Relating Therapy, “At one point [before therapy] I was hearing [voices] for a large proportion of every day—it was almost like—it felt like they were there the whole time,” “It felt like it was constant,” “[now]…when they do come back they're not around for as long—it's literally just a few minutes”

As well as a general reduction in frequency, Jude has noticed that their new-found ability to assert them self has not just increased their sense of power and control, it has significantly reduced the perceived power of their voices, “I feel like the voices don't have as much power anymore”

Jude doesn't sense that the voice's personalities have changed, but they do note that the way they think about them has changed, “the voices personalities haven't changed, really,” “[but] I used to think they were demons, now I'm convinced they're to do with my mental health”

In sum, Jude has noticed significant changes in the frequency of their voices. They also report a reduction in their perceived power, and gained insight into the fact that their voices are not real. It is worth noting that, in line with our predictions, these improvements result in a lowering of the frequency of AVHs, but not in positive changes in either the personalities of the voices, or what the voices say. This is because the very presence of the voices is tied to their negativity and malevolence (their “personalities”), which in turn dictates the linguistic content. We examine the latter now.

### Changes in the linguistic content (“what the voice says”)

Jude was keen to point out that the content of the voices had changed very little. When asked whether the voices say the same kinds of things as they used to [before they accessed Relating Therapy], they responded “yeah, pretty much…they tend to put me down,” “they haven't said all the nasty violent stuff for a while, but they [still] pick up on things that I'm worried or anxious about.”

However, Jude is certain that the very same linguistic content has less of a negative impact on them than it did before Relating Therapy, “they do say a lot of the same things, but it doesn't seem to bother me as much,” “[now] I think I've always had these thoughts about myself, and just because voices are saying them out-loud it doesn't mean it's true, necessarily.”

Again, this is in keeping with our prediction that the likely changes resulting in RT would be circumscribed to the frequency of the voices, and to the negative impact that the voices have on the hearer. There would not be a change in the linguistic content, since the malevolent speaker behind the voice wouldn't say anything positive without thereby becoming a benevolent speaker, and a benevolent speaker is unlikely to be represented in the patient's experience. Put simply, RT does not make negative voices turn into positive voices, since the increased positivity would likely make the voices disappear.

## Ethics statement

This study was carried out in accordance with the recommendations of the Clinical Audit Department of Sussex Partnership NHS Foundation Trust, with written informed consent from the subjects. All subjects gave written informed consent in accordance with the Declaration of Helsinki. The protocol was approved by the Clinical Audit Department at Sussex Partnership NHS Foundation Trust.

## Author contributions

FD: contributed significantly to the ideas underpinning the paper, wrote at least half of the paper, formatted and prepared for submission; MH: contributed to the ideas underpinning the paper, wrote the first two sections of the paper, provided access to the exit interview data discussed.

### Conflict of interest statement

The authors declare that the research was conducted in the absence of any commercial or financial relationships that could be construed as a potential conflict of interest.

## Footnotes

^1^In contrast, others might argue that patients should not be encouraged to integrate their voices, and should be discouraged from viewing their voice as a distinct intentional agent (with his or her own thoughts, feelings etc.) which differ from their own.

^2^Gender neutral pronouns have been used at Jude's request
